# Advancing Understanding of Non-Small Cell Lung Cancer with Multiplexed Antibody-Based Spatial Imaging Technologies

**DOI:** 10.3390/cancers15194797

**Published:** 2023-09-29

**Authors:** Simon Gray, Christian H. Ottensmeier

**Affiliations:** 1 Department of Molecular and Clinical Cancer Medicine, Faculty of Health and Life Sciences, University of Liverpool, Ashton St., Liverpool L69 3GB, UK; 2 Department of Medical Oncology, The Clatterbridge Cancer Centre NHS Foundation Trust, Pembroke Pl., Liverpool L7 8YA, UK

**Keywords:** NSCLC, microenvironment, multiplex, spatial, immunofluorescence, immunohistochemistry, cytometry, immuno-oncology

## Abstract

**Simple Summary:**

Non-small cell lung cancer is common and potentially lethal. Existing treatments that enable a person’s own immune system to attack their cancer significantly improve survival, but only for a minority of people. This difference between people likely depends on the types of cells, secreted molecules and other conditions present in and around the tumour. New technologies have recently allowed many cell types and molecules to be identified on the same tumour slide, whereas previously only a few cell markers could be used; this allows common and rare cell types to be reliably identified, and the relationships between different cells and cell types to be studied in far greater detail than before. These technologies may help to identify new cancer treatments to improve outcomes for patients. Here, we review studies which have used these new technologies in non-small cell lung cancer, and aim to summarise their findings.

**Abstract:**

Non-small cell lung cancer (NSCLC) remains a cause of significant morbidity and mortality, despite significant advances made in its treatment using immune checkpoint inhibitors (ICIs) over the last decade; while a minority experience prolonged responses with ICIs, benefit is limited for most patients. The development of multiplexed antibody-based (MAB) spatial tissue imaging technologies has revolutionised analysis of the tumour microenvironment (TME), enabling identification of a wide range of cell types and subtypes, and analysis of the spatial relationships and interactions between them. Such study has the potential to translate into a greater understanding of treatment susceptibility and resistance, factors influencing prognosis and recurrence risk, and identification of novel therapeutic approaches and rational treatment combinations to improve patient outcomes in the clinic. Herein we review studies that have leveraged MAB technologies to deliver novel insights into the TME of NSCLC.

## 1. Introduction

Lung cancer caused 1.8 million deaths globally in 2020 [1], with non-small cell lung cancer (NSCLC) accounting for around 80–85% of cases [2]. In the United States, approximately half of NSCLC patients present with unresectable or metastatic advanced disease (aNSCLC) [3]. Historically, 30–55% of patients whose disease is resected with curative intent will experience relapse [4]; unresectable, recurrent or metastatic NSCLC without a targetable driver mutation typically carries a poor prognosis, with overall survival (OS) in the range of 15–24 months among patients fit to receive treatment [5,6]. More effective treatments are, thus, sorely needed. Study of the tumour microenvironment (TME) has the potential to deliver insights which may optimise existing treatments for NSCLC as well as aid in the development of novel treatments.

**Figure 1:**
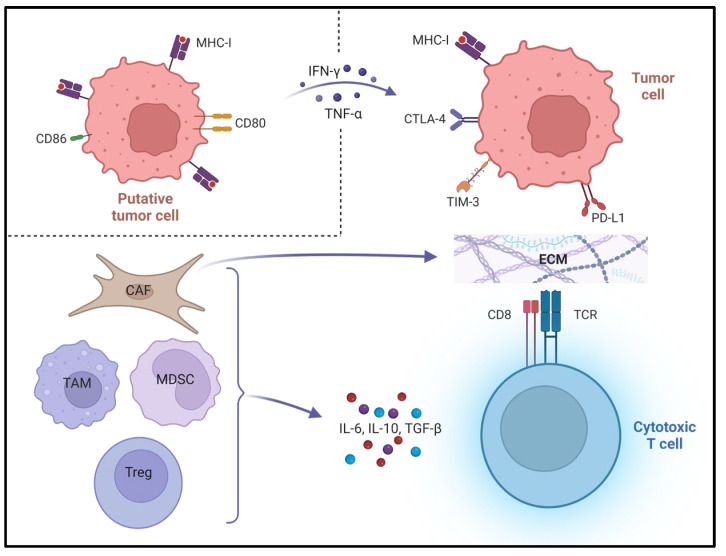
Compromised antigen presentation in the tumour microenvironment. Co-inhibitory and co-stimulatory molecules on cytotoxic T-cell not shown. Figure created with Biorender.com. MHC = major histocompatibility complex. CD = cluster of differentiation. IFN-γ = interferon gamma. TNF-α = tumour necrosis factor alpha. CTLA = cytotoxic T lymphocyte antigen. TIM = T-cell immunoglobulin and mucin domain-containing protein. PD-L1 = programmed death ligand 1. CAF = cancer-associated fibroblast. ECM = extracellular matrix. MDSC = myeloid-derived suppressor cell. TCR = T-cell receptor. IL = interleukin. TGF-β = transforming growth factor beta.

The TME represents an entire ecosystem in which cells of the tumour, stroma, immune system and vasculature co-exist and interact dynamically to affect the course of a putative cancer. The immune system applies competing mechanisms to balance the avoidance of autoimmunity with the ability to deliver robust responses to substances detected as foreign. Successful cancers acquire the ability to tilt the balance of the local immune milieu towards tolerance [7]. Accordingly, multiple immune and stromal cell types are observed to perform both pro- and anti-tumour roles in the TME. Tumours which grow to become clinically detectable are thought to be capable of reducing the activity of anti-tumour cells to support immune escape, tumour progression and metastasis [8,9,10,11]. This is achieved by both direct cell–cell interactions and the release of soluble factors into the TME. Effective antigen presentation (as displayed in Figure 1, according to the 3-signal model) may be compromised in a multitude of ways by the tumour and an immunosuppressive TME [12]. Three tiers of signal are required for full effector function: Signal 1 is engagement of major histocompatibility complex class 1 (MHC-I) on a tumour cell by a cytotoxic T-cell’s T-cell receptor; cancer-associated fibroblasts (CAFs) may produce copious extracellular matrix (ECM) to limit access of cluster of differentiation (CD) 8+ cytotoxic T-cells to tumour cells [13]; myeloid-derived suppressor cells (MDSCs) can release nitrogen species to prevent chemoattractants from recruiting antigen-specific T-cells from the circulation; and MHC-I may be downregulated by tumour cells [14]. Signal 2 is the engagement of co-inhibitory and co-stimulatory receptor/ligand pairs—co-inhibitory molecules include programmed death (/ligand) 1 (PD-[L]1), cytotoxic T lymphocyte antigen 4 (CTLA-4), T-cell immunoglobulin and mucin domain-containing protein 3 (TIM-3) [15]. These are known to be upregulated on tumour cells in response to pro-inflammatory cytokines such as interferon γ (IFN-γ) and tumour necrosis factor α (TNF-α), while co-stimulatory molecules including CD80 and CD86 are downregulated [16,17,18]. Signal 3 comprises the effects mediated by secreted cytokines such as IFN-γ or interleukin 12 (IL-12), and can set the balance between full activation and tolerance [19]. The secretion of immunosuppressive cytokines such as IL-6, IL-10 and transforming growth factor β (TGF-β) by tumour cells, tolerogenic T regulatory (T_reg_) cells, CAFs and tumour-associated macrophages (TAMs) can also influence Signal 3 [20]. Depending on these signals, the functional states of T-cells can range from hyperstimulation to tolerance, anergy and apoptosis [21]. Table 1 serves as a reference for cell surface markers and secreted molecules referred to in the text. 

Methods for interrogating the cellular environment of tissue specimens including classical haematoxylin and eosin staining supplemented by immunohistochemistry (IHC), immunofluorescence (IF), imaging mass cytometry (IMC) and transcriptomics offer a spectrum of resolution with varying depth of tissue analysis and spatial information. For example, traditional IHC preserves 2-dimensional tissue architecture while allowing one or a few markers of interest to be stained per slide [22]. Meanwhile, single cell ribonucleic acid sequencing (RNAseq) can define cells’ transcriptional status in great depth to identify novel subsets and cell trajectory but with loss of information regarding spatial context and cell–cell interaction [23]. Spatial analysis of tumour tissue and the TME is not new, as reflected in the immune-hot/cold/excluded classification of tumours which began in colorectal cancer (CRC) and has shown utility in multiple cancer types [24]. This led to the introduction of the Immunoscore, which prognosticates patients with resected CRC more effectively than pathological tumour or nodal stage, microsatellite instability status, lymphovascular invasion and tumour differentiation despite measuring only two markers (CD3 and CD8) in two locations (tumour centre [TC] and invasive margin [IM]) [25,26]. Despite its remarkable ability to predict relapse status, the Immunoscore has not been adopted in clinical practice [27]. Spatial analyses of haematoxylin/eosin-stained or single-plex IHC-stained tissue continue to be performed using novel artificial intelligence tools [28,29]. In recent years, relevant cell subtypes in the TME have been described which require multiple markers to identify reliably; these include CAF subtypes, T resident memory cells and T follicular regulatory cells [30,31,32]. Obtaining spatial information regarding such subtypes would be extremely challenging with the above technologies. Multiplexed antibody-based (MAB) methods allow spatial analyses to be performed with far greater resolution of cell types and subtypes; such analyses can be performed on the same tissue section, for optimised tissue preservation and analyses of proximity and interaction between cell types. They also generate large volumes of data, which can be challenging to analyse, and a number of open source and commercial image analysis software packages have been developed to facilitate this [33,34].

Attempts to perform multiplexing of markers using brightfield IHC methods are limited by several factors including chromogenic overlap of simultaneously interpreted antibodies, while ‘strip-and-stain’ methods are labour intensive and result in progressive tissue degradation with serial staining rounds [22]. Current state-of-the-art methods for MAB imaging may be classified according to mode of antibody tagging (which may be with fluorophores, deoxyribonucleic acid [DNA] oligonucleotide barcodes or metal tags) and mode of detection (for example fluorescence, chromogen deposition or mass spectrometry). Briefly, MAB techniques feature a multistep process in which protein markers are labelled with fluorescent or tagged antibodies. For example, multiplex immunofluorescence (mIF) involves labelling all protein markers with DNA-barcoded antibodies and then multiple rounds of reaction with a smaller number of complementary fluorescent oligonucleotides, imaging and removal of the fluorescent component. Meanwhile, IMC involves tagging markers with heavy metal-bound antibodies, and application of a highly focused laser which ablates a minute portion of a sample at a given time, with quantification of the metal in the antibody tag using time-of-flight analysis. The details of these methods have been thoroughly reviewed elsewhere [35,36,37]. Typically, 10–60 markers are deployed, though 100-plex panels have been proven feasible using DNA barcode-based methods [38]. Digital spatial profiling (DSP) is notable among these methods as ROIs are selected and indexing oligonucleotides from a given ROI are analysed together [39]. It therefore allows differences between, but not within, ROIs to be analysed and does not preserve spatial information on the entire tissue section.

Immune checkpoint blockade (ICB), particularly with monoclonal antibodies (mAbs) targeting the co-inhibitory PD-(L)1 axis, represents a major breakthrough in the treatment of aNSCLC. Unlike chemotherapy, the historical comparator, ICB can provide long-term responses beyond cessation of treatment [6]. Anti-PD-(L)1 treatment has been FDA-approved for nearly a decade, and may be used as monotherapy or in combination with chemotherapy [40,41]. However, only around 20–30% of patients gain long-term benefit from anti-PD-(L)1 mAbs. These antibodies can further cause severe and long-term toxicity, which is especially relevant as these treatments move into the adjuvant and neoadjuvant settings [42,43,44]. Current tools utilised in clinical practice to predict response to anti-PD-(L)1 therapy include assessments of PD-L1 expression on tumour tissue and within the TME, and tumour mutational burden (TMB) [45,46]. A systematic review and meta-analysis of 8135 patients in 10 solid tumour types compared these methods, as well as gene expression profiling (GEP), with multiplex (m)IHC/mIF [47]. The highest predictive power for benefit from anti-PD-(L)1 therapy was with mIHC/mIF approaches (area under curve [AUC] 0.79) on plotting of summary receiver operating characteristic curves, compared with PD-L1 IHC (AUC 0.65 and *p* < 0.001), GEP (AUC 0.65 and *p* = 0.003) and TMB (AUC 0.69 and *p* = 0.049). This increase in predictive power was achieved using only relatively low-plex mIHC/mIF, with an average of 2–3 markers examined [47]. Study of the NSCLC microenvironment may help answer key clinical questions including identifying predictors of benefit from PD-(L)1 treatment and defining rational combination therapies; herein we present findings from a selection of studies primarily employing MAB methodologies to gain insights into the TME of NSCLC [48,49,50,51].

## 2. Literature Review

### 2.1. Prediction of Recurrence and Survival Following Curative-Intent Resection

Given the availability of tumour tissue following curative-intent resection, multiple studies have used MAB-based methodologies to identify signals associated with post-resection recurrence risk and OS. One relatively early study utilised a 6-plex tyramide signal amplification-based panel to assess 120 patients with resected NSCLC [52]. The cross-G function—a form of probabilistic nearest neighbour analysis—was used to demonstrate shorter OS for patients with CD4+ forkhead box P3-positive (FoxP3+) T_reg_ cells and tumour cells in close proximity (hazard ratio (HR) 1.52, 95% confidence interval [95%CI] 1.11–2.07 and *p* = 0.009). Improved OS for patients with T_reg_ cells and effector CD8+ T-cells (CD8+Ts) in close proximity (HR 0.96, 95%CI 0.92–0.99 and *p* = 0.042) was also demonstrated; the authors suggested this was related to the ability of CD8+Ts to somewhat overcome the tolerogenic effect of T_reg_ cells in the TME [52].

A further paper utilised DSP with a 52-plex panel to study a tumour microarray (TMA) comprised of 92 cases with paired histologically normal adjacent tissue (NAT) [53]. Enrichment of T-cell markers (CD3, CD4), macrophage markers (CD68, CD168), immune checkpoints (CD27 and V-domain Ig suppressor of T-cell activation [VISTA]), CD44 and CD45 were seen in the stroma relative to the tumour. Meanwhile, NAT was enriched with markers of ECM (fibronectin), indoleamine 2,3-dioxigenase 1, exhausted T-cells (lymphocyte activation gene 3 [LAG-3]), TAMs/MDSCs (arginase 1 [ARG-1]), CD34 and the tumour suppressor phosphatase and tensin homolog when compared with the TME [54,55,56,57]. Univariate analysis suggested that expression of CD34, CD3 and inducible co-stimulatory (ICOS) were associated with favourable OS, though these signals did not persist in multivariable analysis with adjustment for age and disease stage [53].

Backman et al., studied 300 patients with resected NSCLC, and used mIF to demonstrate a positive prognostic effect for patients with high densities of tissue helper CD4+ T-cells (CD4+Ts) and CD8+Ts, M1 macrophages, B-cells, plasmacytoid dendritic cells (pDCs) and also both CD4+ and CD8+ T_reg_ cells, including when adjusted for clinical parameters; these observations were stronger when analysing tumour and stromal compartments together [58]. Similar observations were seen between lung adenocarcinoma (LUAD) and lung squamous cell carcinoma (LUSC) samples. In the spatial analysis, helper CD4+Ts and CD8+Ts, M1 macrophages and pDCs were proximal to tumour cells; other immune cell types were more evenly distributed, while mature DCs were predominantly distant from tumour cells. Co-localisation of adaptive lymphocyte subsets together was associated with longer survival, but only the CD8+T/B-cell proximity effect remained significant after multivariate analysis. The positive prognostic impact of CD8+ T_reg_ density was notably abrogated when distance to tumour cells and other immune cell types was accounted for, suggesting high CD8+ T_reg_ density was a reflection of high total immune infiltrate. In assessing relationships between distance and density, multivariable Cox regression analysis showed independently favourable prognosis for high densities of M2-like macrophages, M1 macrophages, close proximity of both CD4+ and CD8+ T_reg_ cells to B-cells, and co-localisation of effector CD8+Ts and tumour cells. Co-localisation of M2 and M1 macrophages conferred a worse prognosis [58]. This group took the commendable decision to make their entire spatial data set publicly available.

Another 2023 analysis by Sorin et al. examined 416 patients with predominantly early-stage LUAD tissue from resection or biopsy via imaging mass cytometry (IMC) of TMAs [59]. Histologically high-grade ‘solid’ tumours were enriched for myeloid cells including tumour-associated neutrophils (TANs), monocytes and CD163+ M2-like macrophages. M2-like macrophages in the ‘solid’ subtype were associated with T_reg_ cells, whereas in other histological subtypes they were strongly correlated with effector CD8+Ts. B-cell frequency was associated with improved survival, independent of a range of potentially confounding clinico-pathologic variables. Spatial analysis of direct cell–cell interaction suggested largely homotypic interactions for tumour cells, endothelial cells and CD163- macrophages; however, tumour cells interacted more with TANs and endothelial cells in higher-grade versus lower-grade histological subtypes. This is consistent with the observed ability of TANs to facilitate haematogenic metastasis [60]. Lower-grade tumours featured greater interaction between tumour cells and both CD8+ and CD4+Ts; meanwhile, though M2-like macrophages and CD8+Ts coexist across tumour grades, their degree of interaction increases as tumour grade increases. Proliferating (Ki-67+) endothelial cells, presumably implicated in hypoxia and angiogenesis, were associated with poor OS and with TAN interactions in high-grade disease. Multiple TAN subsets were identified and hypoxia-inducible factor 1α-positive TANs were associated with worse OS, while total TAN frequency was not. High frequency of 3 cellular neighbourhoods, ‘B-cell-enriched’, ‘lymphoid enriched’ and ‘pan-immune hotspot 1’, were associated with improved OS across histological subtypes. Dissection of B-cell neighbourhoods suggested abrogation of survival advantage when B-cells were proximal to T_reg_ cells, while proximity between B-cells and helper CD4+Ts without T_reg_ enrichment maintained an association with improved survival, independent of the overall prevalence of B-cell and helper CD4+Ts and of histological subtype. Deep learning approaches were utilised and able, in a validation cohort, to predict progression with 94.2% accuracy using spatial analysis of IMC images with lineage markers, as compared with 74.2% with cell frequencies alone. A streamlined panel of 6 markers, together with spatial information to identify cellular neighbourhoods, produced a predictive accuracy of 93.3% for identifying progression [59].

### 2.2. Prediction of Benefit from Immunotherapy

As previously alluded to, another focus of MAB technologies has been the utilisation of both pre- and post-treatment tissue samples to identify novel biomarkers associated with response to treatment.

Several studies from the same research group utilising DSP to study NSCLC TMA tissue have reviewed multiple aspects of response to anti-PD-(L)1 therapy. One such study analysed tissue from 53 patients who received anti-PD-1 mAb monotherapy and had paired pre-treatment samples [61]. After adjustment for clinico-pathologic variables including the serum-based lung immune prognostic index (LIPI) [62], in multivariate analysis only high levels of CD4 and the natural killer cell marker CD56 measured in the immune (CD45+) compartment predicted clinical benefit (partial response or stable disease for ≥6 months), longer progression-free survival (PFS) and OS. High VISTA levels predicted lack of clinical benefit and shorter PFS [61].

A further study of 58 patients with aNSCLC and pre-anti-PD-(L)1 treatment samples studied 71 targets to determine mechanisms of treatment resistance [63]. Expression of the calcium-binding protein S100B was associated with improved OS in all four compartments. Immune stromal CD66b expression by tumour-associated neutrophils (TANs) predicted significantly shorter OS and PFS, as well as progressive disease at 12 and 24 months of therapy. A significant association between immune stromal CD66b expression and ICB resistance was seen in an ICB-treated validation cohort (HR 2.05 and *p* = 0.046) irrespective of pre-treatment serum neutrophil:lymphocyte ratio, but did not predict survival in a further non-ICB-treated cohort (HR 1.67 and *p* = 0.06) [63].

A third study focused on identifying markers of sensitivity to PD-(L)1 blockade, using pre-PD-(L)1 mAb tissue samples from a discovery cohort of 56 patients [64]. Expression of CD44, a positive regulator of PD-L1 in lung cancer, in the tumour compartment was associated with longer PFS in multivariate analysis. Intratumoural CD44 expression was significantly lower versus the immune compartment, and was higher both in patients with LUSC and without baseline liver metastasis [65]. Levels of CD44 determined using quantitative IF were associated, on multivariate analysis, with longer PFS (HR 0.31, 95%CI 0.11–0.87 and *p* = 0.022) and OS (HR 0.29, 95%CI 0.09–0.97 and *p* = 0.038), while stromal CD44 expression did not predict outcomes. In an ICB-treated validation cohort, CD44 levels in the tumour compartment predicted PFS upon multivariate analysis after adjusting for performance status, baseline liver metastasis and LIPI score (HR 0.62, 95%CI 0.40–0.96 and *p* = 0.035). This remained significant after adjusting for the tumour proportion score (TPS) at ≥1% and ≥50% cutoffs. A further ICB-untreated NSCLC cohort showed no prognostic association with CD44 expression. In CD44-high ROIs from both validation cohorts, upregulation of PD-L1, TIM-3, ICOS and CD40 was seen (false discovery rate (FDR)-adjusted *p* < 0.05), with other immune cell markers and co-inhibitory molecules upregulated to a lesser extent [64].

A further group studied pre-treatment tissue from 18 patients with aNSCLC and progression through first-line chemotherapy and were enrolled in a Phase 2 clinical trial of a bispecific PD-L1/CTLA-4 antibody [66]. Upon DSP, the stroma featured higher expression of immune cells markers (CD45, CD3, CD8, CD4 and CD11c) compared with tumour, consistent with parallel messenger RNA analysis. Co-clustering was observed between geographically and molecularly similar ROIs, for both tumour and stromal regions. The spatially resolved signature for stroma, versus tumour, was found to be more relevant for survival (AUC 0.838 vs. 0.786, respectively) and was associated with longer OS (*p* = 0.039) with stronger clinical relevance than PD-L1 TPS or TMB. This spatially resolved 18-protein stromal signature was validated in 65 NSCLC samples from patients who had received ICB, with an AUC of 0.776 and significant associations with OS and PFS, emphasizing the importance of the stromal compartment in affecting patient outcomes [66].

### 2.3. Study of CD8+ T-Cells in Early-Stage Resected NSCLC

Among the primary effectors of anticancer immunity are CD8+Ts, which are also potentiated using current ICB strategies [67]; they have, accordingly, been the focus of multiple studies employing MAB methods to study the TME.

One such study identified an exhausted CD8+ T-cell subset using IMC to study resected tumour and paired NAT from 25 early-stage NSCLC patients [68]. The ratio of lymphoid to non-lymphoid cells was significantly higher in tumour versus NAT; CD8+Ts in the tumour were substantially more proliferative versus those in NAT, while other lymphoid cell types were not. Hierarchical clustering divided tumour-associated CD8+Ts into predominantly effector (CD45RA+CD45RO-) and effector memory (CD45RA-CD45RO+) subtypes. The latter was further subdivided into a conventional memory phenotype, while the other expressed high levels of CD45RO, eomesodermin, FAS, CD27, CD28, PD-1, LAG-3 and TIM-3 as well as low T-bet and granzyme B (GZMB), suggestive of a burned-out effector (E_bo_) subset. Such E_bo_ clusters were primarily tumour-associated, while CD8+Ts in NAT showed preserved effector functions. Subsequent CD8+T whole-transcriptome RNAseq confirmed enrichment of apoptotic and dysfunctional CD8+Ts among E_bo_ cells. Anti-PD-1 therapy of NSCLC-engrafted mice demonstrated a post-treatment reduction in the E_bo_ subset while preserving effector CD8+Ts. Subsequent IMC of a mixed-stage human NSCLC cohort showed a higher proportion of CD8+Ts were E_bo_ cells in patients with late-stage, versus early-stage, disease (*p* = 0.006), suggesting expansion with time and disease progression. Furthermore, E_bo_ predominance among CD8+Ts was associated with worse OS (HR 2.66, 95%CI 1.17–6.01 and *p* = 0.03) among patients who received anti-PD-1 therapy [68].

A further study of tissue from 13 treatment-naïve patients with resectable NSCLC used cytometry by time-of-flight and IMC to describe a population of CD8+PD-L1+ tumour-infiltrating T-cells with low levels of expression of PD-1, CD103, GZMB and IFN-γ [69]. Cellular neighbourhood analysis demonstrated close proximity of CD8+PD-L1+ cells to activated and exhausted CD8+Ts, suggesting a regulatory role for the former subset which was subsequently corroborated with demonstration of their capacity to suppress CD8+PD-L1- cells’ production of IFN-γ and TNF-α in vitro [69].

A larger study of 279 resected NSCLC cases was assessed using mIF of TMA sections, specifically seeking associations with lymph node metastasis (LNM) [70]. Density of CD8+Ts was significantly lower in both TC (*p* < 0.001) and IM (*p* < 0.001) for patients with LNM; upon CD8+T subtyping, the densities of pre-dysfunctional (odds ratio (OR) 0.51, 95%CI 0.29–0.88 and *p* = 0.015) and dysfunctional (OR 5.80, 95%CI 3.19–10.54 and *p* < 0.001) CD8+Ts were both associated with LNM, independent of a group of clinico-pathologic factors. Lower recurrence risk was predicted using total CD8+Ts in the IM (HR 0.57, 95%CI 0.35–0.92 and *p* = 0.021), pre-dysfunctional CD8+Ts in the TC (HR 0.55, 95%CI 0.34–0.89 and *p* = 0.014), while dysfunctional CD8+Ts in the IM predicted higher recurrence risk (HR 2.49, 95%CI 1.60–4.13 and *p* = 0.012). Among patients with LNM, mean nearest neighbour distance (mNND) of CD8+Ts was shorter to helper CD4+Ts, T_reg_ cells, CAFs and cancer microvessels (CMVs)—these remained significant after multivariate analysis. Furthermore, shorter mNND was associated with recurrence for CD8+T/T_reg_ in the IM (HR 1.72, 95%CI 1.26–2.92 and *p* = 0.024) and CD8+T/CAF in the IM (HR 1.57, 95%CI 1.11–2.43 and *p* = 0.024) after multivariate analysis. As T stage increased, mNND decreased between CD8+T/CD4+T subsets. Trafficking of CD8+Ts, but not CD4+Ts, into the IM appeared to be impeded by CMVs, as dysfunctional CD8+Ts in the IM were associated with CMVs. Tumour-proximal CD8+Ts were also negatively associated with CAFs, in both the TC and IM, suggesting a further CAF barrier preventing T-cells from accessing a tumour [70].

In comparison to previously considering all CD8+Ts together, studies such as those above clearly show the value of MAB spatial dissection of this cellular compartment in detecting subsets with divergent functions [71]. This is likely to enable prognostic and predictive tools to be refined, and may enable the development of more personalised anticancer agents. While CD8+Ts have historically been synonymous with ‘cytotoxic’ T-cells, their roles in the TME are shown to be far less straightforward.

### 2.4. Other Multiplexed Antibody-Based Studies

An IMC-based analysis of resection specimens from 12 patients with LUSC identified a novel population of CD3-CD4+FoxP3+CD25-CD127- cells in both tumour and adjacent regions of 10 patients, which were also TNF-α-positive and IFN-γ-negative. A pro-inflammatory function, divergent from that of T_reg_ cells, was proposed in view of their TNF-α production, and negativity for CD127 also indicated they were distinct from innate lymphoid cells [72]. A CD3-CD4+CD127+ population was previously identified in autoimmune diseases such as rheumatoid arthritis and psoriasis; despite its T-cell lineage, this was activated by innate signals such as IL-7, which can downregulate CD127 expression in CD3-CD4+ cells [73,74].

A DSP-based study of a TMA formed from 33 patients’ surgically resected NSCLC tissue focused on leucocyte populations in the stroma, tumour or tertiary lymphoid structures (TLS) [75]. Versus stroma, intratumoural lymphocytes expressed higher levels of multiple molecules including PD-L2, CTLA-4 and FoxP3, indicating active immune suppression. In the stroma, fibroblast activation markers were observed, as well as significantly higher VISTA and CD27 expression versus within the tumour. Actively proliferating T- and B-cells were observed more frequently in the TLS versus stroma, with increased CD3, CD20, CD45, beta-2-microglobulin, CD11c, CD40, ICOS and Ki-67. Shorter distance from ROI to tumour was associated with increased expression of immunosuppressive molecules. Expression of co-stimulatory CD27 decreased with proximity to the tumour but was significantly expressed in stromal regions, and a CD27 agonist such as varlilumab was suggested by the authors as a potential means to exploit this. A strong correlation was observed between expression of ARG-1 and CD66b. Given prior observations of ARG-1 production by TANs and ARG-1 blockade reducing tumour growth in an animal model of NSCLC, the authors suggested that tumours with highly frequent granulocytes in either tumour or stroma could be targeted with ARG-1-blocking therapy [75,76,77].

A recent paper by Parra et al. employed 5 mIF panels in parallel, with 6–7 markers per panel, to evaluate TMA sections from 225 patients with resected stage I-III NSCLC [78]. Overall, LUSC samples contained a greater proportion of immune checkpoint-expressing cells than in LUAD, and higher densities of B7-H3 and B7-H4 were noted in LUSC versus LUAD (*p* < 0.001), whereas LUAD samples contained a higher density of IDO-1 (*p* = 0.015). Regarding CD3+ cellular densities, among LUAD higher figures for CD8+, CD45RO+, CD8+CD45RO+, IDO-1+ and TIM-3+ were noted. Higher densities of myeloid DCs and CD66b+ TANs were seen among LUSC samples. In the spatial analysis, tumour cells proximal to T-cells expressed more immune checkpoint molecules, while tumour cells closer to CD68+ macrophages and PD-L1+ macrophages were more likely to be PD-L1-. In LUAD, multiple T-cell subsets (CD8+, CD45RO+, CD8+CD45RO+ and CD4+FoxP3+) showed closer proximity to tumour cells than in LUSC, which exhibited closer distances from tumour cells to T-cells expressing LAG-3, OX40 and TIM-3, and B-cells expressing OX40 and LAG-3. Median distance from tumour cells was cross-referenced with the distribution of immune cell subsets within samples (heterogeneous or clustered). Tumours with heterogeneously distributed immune cells and long distance to tumour cells contained higher densities of both effector and regulatory cells and were considered immune-hot. Meanwhile, tumours featuring clustered immune cells with long distances to tumour cells featured lower immune cell densities and were aligned with an immune-cold/excluded phenotype. In addition to associating spatial features with a variety of clinico-pathological features, significant associations with OS were made. Specifically, after multivariate analysis longer OS was found for close proximity to tumour cells of CD3+, CD3+CD8+GZMB+, CD3+CD8+CD45RO+, CD3+TIM-3+, CD3+ICOS+, CD66b+CD11b+ and CD20+ cells; longer OS was also seen for longer distance to tumour cells of CD3+CD8-FoxP3+, CD3+CD45RO+FoxP3+, CD3+CD8+PD-L1+, CD3+OX40+, LAG-3+ and CD68+PD-L1+ cells [78].

## 3. Discussion

As demonstrated above, a number of potential biomarkers and signatures have been identified pertaining to prognosis, risk of recurrence and prediction of benefit from ICB. While single markers could conceivably be incorporated into existing pathology workflows, other signatures may require MAB equipment for clinical application similar to that used for discovery; this may be due to a large number of included markers, or the integration of spatial information into the signature. Slow sample processing times and high cost currently represent barriers to clinical application of MAB technologies, though the opportunities and challenges of clinically implementing mIF technology have been reviewed elsewhere [79]. Prospective clinical validation of such signatures, while challenging, is likely to be crucial in evidencing the clinical benefits of MAB technologies given the potential cost of such a transition. Artificial intelligence tools have the capacity to effectively leverage the large quantities of data produced using MAB technologies [59], and are likely to feature increasingly in studies thereof. The progressive expansion and validation of larger marker panels, and the expansion of functionality within commercially available image analysis software, are also likely to expand the utility and scope of MAB-based research with time [33,38].

As illustrated in Table 2, the studies presented herein describe a range of results with relatively little replication of key findings between studies. There are many sources of potential variation between studies. Patient and disease characteristics of the studied population may vary regarding histological type and subtype, treatment status and disease stage. Sources of potential variation related to data acquisition include selection of multiplexing method, protein marker panel and antibody clones. Some studies elect to utilise multiple smaller antibody panels while others use a single larger panel, influencing downstream analysis. Once images are acquired, the process of quality control (QC) and setting positivity thresholds for individual markers is not standardised, and multiplexing platforms show a degree of variation in staining between samples. As demonstrated herein, there is wide variation in the process of data analysis, and particularly in spatial analysis. The quantity of cell types and distances to be analysed makes adjustment for multiple comparisons challenging and there is variation in how this is addressed. While validation sets are valuable for confirming findings of apparent statistical significance, having both discovery and validation sets undergo the same QC process, which is distinct from other studies, may produce discordant results between studies.

An inherent limitation of MAB methods, especially utilising TMAs, is tumour heterogeneity and the possibility of non-representative sampling. Where this has been studied in NSCLC, the results have been largely reassuring; in one study, 60 patients who had two TMA cores taken per patient showed 91.7% agreement in predictive outputs between their respective cores [59]. Given many MAB technologies can require the prohibitive amounts of time and expense of analysing large numbers of full biopsy sections, use of TMAs seems a reasonable compromise to allow the study of large numbers of patients. Many MAB studies within NSCLC have focused on earlier-stage, single-site disease; as such, samples are likely to be more representative of the cancer as a whole, given overall tumour burden is lower and heterogeneity between metastases does not have to be contended with. While such issues are inherent in any analysis based on solid biopsy material, MAB technologies may be more limited in the study of metastatic disease for this reason, assuming only one site is sampled.

The authors also note that some recently developed methods of performing spatial analysis fall outside the scope of this review. Methods such as in situ RNAseq [80] and matrix-assisted laser desorption/ionization time-of-flight mass spectrometry [81] offer alternative means to gain spatial information regarding the TME, and indeed have been utilised in the study of NSCLC [82,83]. Spatial proteomics and transcriptomics can provide complementary information, with the latter enabling more accurate cell clustering and enhancing discovery potential, particularly in the case of in situ whole-trancriptome RNAseq where discovery is not limited to positivity with regard to markers from a pre-determined panel [84]. While some included studies have used complementary transcriptomic methods to validate or further investigate findings from MAB technologies, the integration of spatial proteomics and transcriptomics is likely to represent the next step in the molecular characterisation of cell types and states in the TME [71,84].

## 4. Conclusions

Multiplexed antibody-based spatial technologies continue to demonstrate their utility as a novel research tool. Moreover, techniques for analysing the data generated using these techniques continue to advance. Beyond tools for prognostication and prediction of benefit from existing standard-of-care treatments, MAB spatial technologies offer the possibility of identifying TME-based indications for additional therapeutics to overcome treatment resistance. Separation of bystander and driver events, and identifying redundancy, remain challenging but MAB technologies represent a powerful tool for deepening our understanding of the TME. In time, this may enable more rational stratification of patients into appropriate clinical trials, identifying therapies which may only benefit patients with a certain TME status.

## Figures and Tables

**Table 1:** Expression patterns of included cell surface markers and functional classification of included secreted molecules.

Widely Expressed Cell Surface Markers				
FAS	MHC-I			
**T-cell markers**				
CD3	CD4	CD8	CD45RA	CD45RO
CD103	CD127	FoxP3	LAG-3	
**Co-inhibitory molecules**				
B7-H3	B7-H4	CTLA-4	IDO-1	PD-1
PD-L1	PD-L2	TIM-3	VISTA	
**Co-stimulatory molecules**				
CD27	CD28	CD40	CD80	CD86
ICOS	OX40			
**M2-like macrophage markers**				
CD163	CD168			
**Secreted pro-inflammatory molecules**				
IFN-γ	TNF-α	GZMB		
**Secreted tolerance-promoting molecules**				
IL-6	IL-10	TGF-β	ARG-1	
**Other immune cell markers**				
CD11b (myeloid cells; NK cells)		CD11c (DCs; NK cells; activated T- or B-cells)		
CD25 (DCs)		CD45 (pan-leucocyte)		CD56 (NK cells)
CD66b (TANs)		CD68 (pan-macrophage)		
**Other cell surface markers**				
CD34 (CAFs; endothelial cells)		CD44 (CSCs; CAFs)		
Cytokeratins (tumour cells)		Ki-67 (proliferating cells)		

MHC = major histocompatibility complex. CD = cluster of differentiation. FoxP3 = forkhead box P3. CTLA = cytotoxic T-lymphocyte antigen. IDO = indoleamine 2,3-dioxigenase. PD-(L)1 = programmed death (ligand) 1. TIM = T-cell immunoglobulin and mucin domain-containing protein. VISTA = V-domain Ig suppressor of T-cell activation. ICOS = Inducible co-stimulator. TAN = tumour-associated neutrophil. DC = dendritic cell. NK = natural killer. CAF = cancer-associated fibroblast. CSC = cancer stem cells. LAG = lymphocyte activation gene. IFN-γ = interferon gamma. TNF-α = tumour necrosis factor alpha. GZMB = granzyme B. TGF-β = transforming growth factor beta. ARG = arginase.

**Table 2:** Summary of selected clinically relevant findings from included studies.

Reference; Multiplexing Method	Setting	Finding	Outcome Predicted	Reported Measure of Predictive Value
Barua et al. [52]; TSA	Post curative-intent resection	Tumour cell/T-reg interactions	Inferior OS	Greater AUC of cross-G function: HR 1.52; 95%CI 1.11–2.07, *p* = 0.009
		CD8+T/T-reg interactions	Superior OS	Greater AUC of cross-G function: HR 0.96; 95%CI 0.92–0.99, *p* = 0.042
Backman et al. [58]; mIF	Post curative-intent resection	Greater CD8+ effector/ tumour cell proximity	Superior OS	Stepwise Cox regression: HR 0.29, *p* <0.05
		Greater M2 macrophage/ M1 macrophage proximity	Inferior OS	Stepwise Cox regression: HR 2.33, *p* <0.05
		Greater B-cell/ CD4+ T-reg proximity	Superior OS	Stepwise Cox regression: HR 0.59, *p* <0.05
		Greater CD8+ T-reg/ B-cell proximity	Superior OS	Stepwise Cox regression: HR 0.46, *p* <0.01
Sorin et al. [59]; IMC	Adenocarcinoma (mixed-stage)	Frequent ‘B-cell-enriched’ CNs	Superior OS	Log-rank test: *p* = 0.001
		Frequent ‘lymphoid enriched’ CNs	Superior OS	Log-rank test: *p* = 0.039
		Frequent ‘pan-immune hotspot 1’ CNs	Superior OS	Log-rank test: *p* = 0.026
		Frequent ‘undefined’ CNs	Inferior OS	Log-rank test: *p* = 0.006
	Adenocarcinoma (stage I)	Deep learning signature lineage marker model	Recurrence	Prediction score: 95.9% (vs 80.55% with clinical variables), *p* = 0.0343. Validation cohort: Accuracy 94.2% (vs 75% baseline prediction score).
Zugazagoitia et al. [61]; DSP	ICI-treated (stage III-IV)	Frequent CD56+ cells in the leucocyte compartment	Superior PFS	Log-rank test: HR 0.24, *p* = 0.006
			Superior OS	Log-rank test: HR 0.26, *p* = 0.014
		Frequent CD4+ cells in the leucocyte compartment	Superior PFS	Log-rank test: HR 0.31, *p* = 0.006
			Superior OS	Log-rank test: HR 0.23, *p* = 0.0.007
Moutafi et al. [63]; DSP	ICI-treated (advanced-stage)	Frequent CD66b+ cells in the leucocyte compartment	Inferior OS	Log-rank test: HR 1.31, *p* = 0.016
Moutafi et al. [64]; DSP	ICI-treated (advanced-stage)	Frequent CD44+ cells in the tumour compartment	Superior PFS	Discovery cohort: log-rank test HR 0.68, *p* = 0.043 Validation cohort: log-rank test: HR 0.62, *p* = 0.03
Song et al. [66]; DSP	ICI bispecific Ab-treated (advanced-stage); validation set was ICI mAb-treated	Stromal signature	Treatment response	Training set: AUROC 0.838 Validation set: AUROC 0.776
			Superior PFS	Log-rank test: HR 2.90, *p* = 0.013 in validation set
			Superior OS	Validation set: log-rank test HR 3.44, *p* = 0.04
		Tumour signature	Treatment response	Training set: AUROC 0.786
Gerdtsson et al. [67]; DSP	Post curative-intent resection	High B7-H3 expression	Superior OS	Log-rank test: HR 0.60, *p* = 0.008
Sanmamed et al. [71]; IMC, mIF	Post curative-intent resection	Low proportion of CD8+ T-cells with ‘E_bo_’ phenotype	Durable clinical benefit	Student’s *t*-test: *p* < 0.001
	ICI-treated (advanced-stage)	Low proportion of CD8+ T-cells with ‘E_bo_’ phenotype	Superior OS	Log-rank test: HR 2.66, 95%CI 1.17–6.01, *p* = 0.03
Yang et al. [73]; mIF	Post curative-intent resection	Low density of ‘pre-dysfunctional’ CD8+ T-cells in tumour centre	Superior RFS	Log-rank test: HR 0.55, 95%CI 0.34–0.89; *p* = 0.014
		High density of ‘dysfunctional’ CD8+ T-cells in invasive margin	Inferior RFS	Log-rank test: HR 2.49, 95%CI 1.60–4.13; *p* = 0.012
		Shorter mNND between CD8+ T-cells and T-regs in invasive margin	Inferior RFS	Log-rank test: HR 1.72, 95%CI 1.26–2.92; *p* = 0.024
		Shorter mNND between CD8+ T-cells and CAFs in invasive margin	Inferior RFS	Log-rank test: HR 1.57, 95%CI 1.11–2.43; *p* = 0.024
Parra et al. [78]; mIF	Adenocarcinoma post curative-intent resection	Greater CD66b+ cell/ tumour cell proximity	Superior RFS	Log-rank test: *p* = 0.028
		Greater CD3+CD8+ cell/ tumour cell proximity	Superior RFS	Log-rank test: *p* = 0.041
		Greater CD68+ cell/ tumour cell proximity	Superior RFS	Log-rank test: *p* = 0.021
		Greater CD3+B7-H3+ cell/ tumour cell proximity	Inferior RFS	Log-rank test: *p* = 0.022
	Squamous cell carcinoma post curative-intent resection	Greater CD3+PD-L1+ cell/ tumour cell proximity	Inferior RFS	Log-rank test: *p* = 0.012
		Greater CD3+ICOS+ T-cell/ tumour cell proximity	Superior RFS	Log-rank test: *p* = 0.009

All patients in the ‘post curative-intent resection’ setting did not receive neoadjuvant therapy. Univariate associations which did not persist in multivariate analysis are not included. ‘Advanced-stage‘ refers to unresectable, recurrent or metastatic non-small cell lung cancer. TSA = tyramide signal amplification. OS = overall survival. AUC = area under curve. CD = cluster of differentiation. HR = hazard ratio. mIF = multiplex immunofluorescence. IMC = imaging mass cytometry. CN = cellular neighbourhood. DSP = digital spatial profiling. ICI = immune checkpoint inhibitor. PFS = progression-free survival. mAb = monoclonal antibody. AUROC = area under receiver operating curve. Ebo = burned out effector. RFS = recurrence-free survival. mNND = mean nearest neighbour distance. PD-L1 = programmed death ligand 1. ICOS = inducible co-stimulator.
